# The Different Impact of ERK Inhibition on Neuroblastoma, Astrocytoma, and Rhabdomyosarcoma Cell Differentiation

**DOI:** 10.32607/actanaturae.11461

**Published:** 2021

**Authors:** T. D. Lebedev, E. R. Vagapova, V. S. Prassolov

**Affiliations:** Engelhardt Institute of Molecular Biology, Russian Academy of Sciences, Moscow, 119991 Russia

**Keywords:** cell differentiation, malignant tumors, ERK inhibitors, growth factors, fluorescent reporter

## Abstract

Aberrant ERK activity can lead to uncontrolled cell proliferation, immortalization, and impaired cell differentiation. Impairment of normal cell differentiation is one of the critical stages in malignant cell transformation. In this study, we investigated a relationship between ERK tyrosine kinase activity and the main differentiation features (changes in cell morphology and expression of genes encoding differentiation markers and growth factor receptors) in SH-SY5Y neuroblastoma, U-251 astrocytoma, and TE-671 rhabdomyosarcoma cells. ERK activity was assessed using a reporter system that enabled live measurements of ERK activity in single cells. We demonstrated that suppression of ERK activity by selective ERK inhibitors, in contrast to a commonly used differentiation inducer, retinoic acid, leads to significant changes in TE-671 cell morphology and expression of the myogenic differentiation marker genes PROM1, MYOG, and PAX7. There was a relationship between ERK activity and morphological changes at an individual cell level. In this case, SH-SY5Y cell differentiation induced by retinoic acid was ERK-independent. We showed that ERK inhibition increases the sensitivity of TE-671 cells to the EGF, IGF-1, and NGF growth factors, presumably by reducing basal ERK activity, and to the BDNF growth factor, by increasing expression of the TrkB receptor.

## INTRODUCTION

Extracellular signal-regulated kinases 1/2 (ERK1/2) play a key role in important processes such as cell proliferation, survival, and differentiation [1, 2]. In this case, the effect of ERK activation on these processes often depends on the cell type, activation signal and its duration, and the dynamics of ERK activity, which significantly complicates the identification of the specific role of ERK in cellular processes. Usually, ERK activation is associated with cell survival and proliferation signals [3]. However, depending on the cell type, ERK inhibition can both stimulate and prevent cell death [4].

The emergence of various reporter systems to monitor ERK activity in living cells has stimulated research in this area [5, 6, 7, 8, 9]. However, there is no generally accepted model describing the effect of ERK on cell differentiation. ERK is known to directly inhibit the activity of pluripotency-associated transcription factors, such as NANOG, OCT4, KLF2, and KLF4 [10, 11]. Downregulation of ERK activity, e.g., by MEK inhibitors, stimulates the self-renewal of embryonic stem cells via the inhibition of ERK-dependent differentiation [12]. However, in some cases, ERK inhibition stimulates cell differentiation, in particular in neuroectoderm cells or bone marrow mesenchymal stem cells [13, 14]. Many growth factors, such as FGF, NGF, PDGF, BDNF, EGF, and IGF-1, play an important role in cell differentiation [15, 16]. Certain growth factors controlling survival of differentiated cells are often essential in the late stages of differentiation. In this case, many growth factors act through ERK activation. Therefore, ERK activation can differently affect differentiation, depending on the stage and cell type.

ERK activity is upregulated in most malignant tumors, in particular due to activating mutations in the MAPK signaling cascade. In this case, activating mutations in the RAS genes inhibit epidermal cell differentiation [17, 18, 19]. Investigation of malignant cell differentiation is required to understand malignant cell transformation and develop approaches to tumor therapy. For example, approaches based on retinoic acid-stimulated cell differentiation are used in the therapy of neuroblastomas [20] and some types of leukemia [21]. In addition, inhibition of the RAS-MEK-ERK signaling cascade is considered a promising approach to the treatment of rhabdomyosarcomas, astrocytomas, and neuroblastomas [22, 23, 24].

When testing the effectiveness of ERK inhibitors in various cells, we noticed morphological changes in some cell types, which were similar to the changes associated with differentiation. In this study, we used a reporter system enabling measurements of ERK activity in live single cells to quantify the relationship between ERK activity and differentiation of various malignant cells.

## EXPERIMENTAL

**Cell cultures and reagents**

Continuous TE-671 rhabdomyosarcoma and U-251 astrocytoma cells as well as HEK293T embryonic kidney cells were cultured in a DMEM medium (Gibco, USA). SH-SY5Y neuroblastoma cells were cultured in a RPMI-1640 medium (Gibco) containing 10% fetal bovine serum (FBS), 100 U/mL penicillin, 100 μg/mL streptomycin, 1 mM sodium pyruvate, and 2 mM L-glutamine at 37°C and 5% CO_2_. All cell lines were donated by the Heinrich-Pette Institute – Leibniz Institute for Experimental Virology (Hamburg, Germany). We used all-trans retinoic acid (R2625) and Hoechst 33342 DNA dye (14533) (Sigma-Aldrich, USA). We used SCH772984 (S7101), Ulixertinib (S7854), and VX-11e (S7709) (Selleckchem, USA) ERK inhibitors. All reagents were initially diluted in DMSO. We also used recombinant human growth factors EGF (ab179628), IGF-1 (ab9573), NGF (ab179616), and BDNF (ab206642) (Abcam, UK).

**Production of ERK-KTR reporter cell lines**

Lentiviral particles directing expression of the gene encoding the ERK-KTR reporter protein were prepared by calcium phosphate transfection of HEK293T cells using a ProFection® Mammalian Transfection System kit (Promega, USA, E1200). We used pMDLg/pRRE and pRSV-Rev third-generation packaging plasmids and a plasmid encoding the VSV-G coat protein. The pLentiCMV Puro DEST ERKKTRClover lentiviral vector was received from Addgene (#59150). After lentiviral transduction, TE-671, SH-SY5Y, and U-251 cells were selected with puromycin (Sigma-Aldrich, P7255) until more than 80% of the cells were positive for the reporter protein. After lentiviral transduction, the TE-671, SH-SY5Y, and U-251 cells were selected on media containing puromycin (0.5–2 μg/mL), which provided a population where more than 80% of the cells were reporter-protein positive.

**Processing of cell images and calculations of ERK activity and cell length**

Cell Images were acquired using a Leica DMI8 automated fluorescence microscope (Germany). Images were processed using the CellProfiler 4 software. Segmentation of Hoechst 33342-stained nuclei was assessed using the Otsu image thresholding algorithm. Cytoplasmic boundaries were determined based on the fluorescent ERK-KTR reporter signal using the position of nuclei to evaluate cell boundaries by the Sauvola image thresholding algorithm. To calculate the lengths of the cytoskeleton and cell processes, the cell body was first defined (a 3- to 5-pixel radius around the nucleus), and then the cytoskeleton was binarized based on a fluorescent reporter signal. Binarization parameters were selected for each cell type at all stages. Incorrectly recognized cells, elimination of outliers and artifacts, and subsequent data processing were performed using original algorithms in Python 3.8. The protocols used for CellProfiler are available at: https://github.com/CancerCellBiology/ActaNaturae-2021.

**Assessment of gene expression**

**Table 1:** Primers used in real-time PCR

Primer	Nucleotide sequence 5’→3’
GAPDH pr1	GAGCCCGCAGCCTCCCGCT
GAPDH pr2	GCGCCCAATACGACCAAATC
PROM1 pr1	CCTGGTCCAACAGGGCTATC
PROM1 pr2	TCGTGGTTTGGCGTTGTACT
RBFOX3 pr1	CAGACAGTGCCGCAGACAG
RBFOX3 pr2	TTCTCTGTAGGGTCGGAGGG
TUBB3 pr1	ATGAGCATGGCATCGACCC
TUBB3 pr2	AGGCACGTACTTGTGAGAAGA
MYOG pr1	TCAGCTCCCTCAACCAGGAG
MYOG pr2	CCGTGAGCAGATGATCCCC
PAX7 pr1	CACTGTGACCGAAGCACTGT
PAX7 pr2	TCCAGCCGGTTCCCTTTGT
EGFR pr1	AGGAGAGGAGAACTGCCAGAA
EGFR pr2	TCTCGGAATTTGCGGCAGAC
IGF1R pr1	CATCCGACGGGGGAATAACA
IGF1R pr2	GCTGCAAGTTCTGGTTGTCG
NTRK1 pr1	CCATCCCTGACACTAACAGCA
NTRK1 pr2	GCACAAGGAGCAGCGTAGAA
NTRK2 pr1	CTGAACCAAGCACGGTTTCC
NTRK2 pr2	CAGGGGCAGAAACTCCAGAA

Total RNA was isolated by chloroform-trizol extraction using the TRIzol reagent (Thermo Scientific, USA, 15596018) according to the manufacturer’s protocol. Total RNA (1 μg) was used to prepare cDNA using a RevertAid First Strand cDNA Synthesis Kit (Thermo Scientific, K1622), according to the manufacturer’s protocol. Expression was analyzed by real-time PCR using a qPCRmix-HS SYBR kit (Evrogen, Russia, PK147L) on a Bio-Rad CFX96 device (USA). Results were processed using the Bio-Rad CFX Manager 3.1 and GraphPad Prism 9.1 software. A list of primers is shown in the Table.

## RESULTS

**Creation of ERK-KTR reporter expressing cell lines**

The role of ERK in cell differentiation was studied in three lines of malignant cells capable of in vitro differentiation: SH-SY5Y neuroblastoma, U-251 MG astrocytoma, and TE-671 rhabdomyosarcoma cells. Lentiviral transduction of cells of these lines resulted in cells expressing the ERK activity reporter, ERK-KTR, a chimeric protein composed of the ERK1/2 docking site of the ELK1 protein, nuclear localization signal (NLS), nuclear export signal (NES), and green fluorescent protein mClover [25]. In contrast to NES, NLS in the chimeric protein is activated, which ensures predominantly nuclear localization of the reporter protein. Activated ERK1/2 kinases occur in the cell nucleus, where they bind to the ELK1 docking site and phosphorylate the NLS and NES regions. This activates the nuclear export signal and deactivates the nuclear localization signal, which leads to translocation of the reporter protein from the nucleus to the cytoplasm. In the cytoplasm, the reporter protein is dephosphorylated by cellular phosphatases and transferred back to the nucleus. The reporter protein distribution between the nucleus and cytoplasm is established depending on an ERK activity level. The fluorescent protein in the reporter enables an evaluation of ERK activity by the ratio of the fluorescent protein signal intensity in the cytoplasm and the nucleus. Thus, the ERK-KTR reporter provides an evaluation of ERK activity in live individual cells using a fluorescence microscope.

**Fig. 1:**
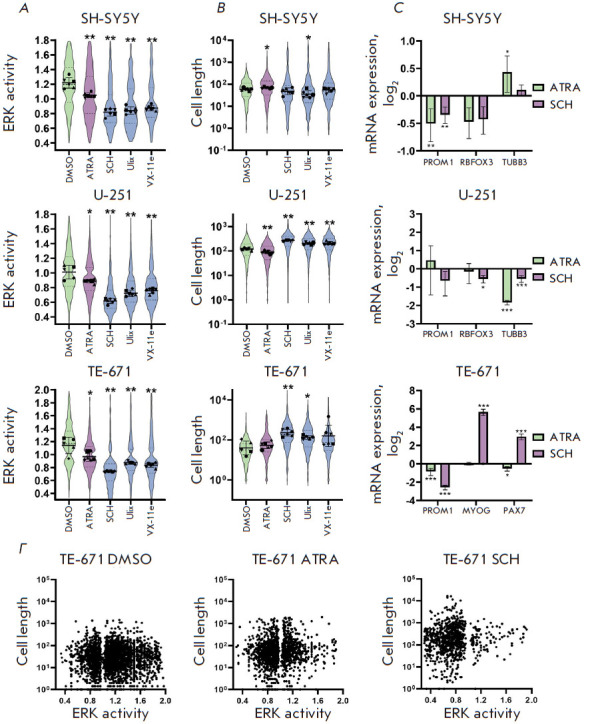
SH-SY5Y, U-251, and TE671 cells with ERK-KTR reporter expression. (*A*) – Images of SH-SY5Y, U-251, and TE671 cells with ERK-KTR reporter expression 72 h after addition of 10 μM retinoic acid (ATRA) or 250 nM of the ERK inhibitor SCH772984 (SCH). (*B*) – Examples of cell images processed using the CellProfiler 4 software. Images are shown in a gray gradient. The cytoplasm boundaries are marked in green. The nuclei are marked in blue; the nuclei were identified by staining with the Hoechst 33342 DNA dye

Next, we treated ERK-KTR expressing cells with ERK inhibitors and all-trans retinoic acid (ATRA) that is widely used for induction of differentiation of various cell types and for neuroblastoma therapy. For the initial test, we selected three ERK inhibitors that had been effective in clinical trials: SCH772984, Ulixertinib, and VX-11e. Cells were treated with ERK inhibitors (250 nM) or ATRA (10 μM) for 72 h, and images were acquired on a fluorescence microscope. All inhibitors significantly reduced the ERK activity, which is evident from the changes in the fluorescent signal distribution in the nucleus and the cytoplasm (Fig. 1A). We also noticed morphological changes in SH-SY5Y cells induced by ATRA, as well as in TE-671 and U-251 cells induced by ERK inhibitors. The most pronounced changes were caused by SCH772984 (Fig. 1A). The observed morphological changes included elongation of the cell processes and the entire cytoskeleton, especially in TE-671 cells. These changes are similar to the previously reported morphological changes characteristic of cell differentiation. To quantify the observed changes, we developed algorithms for the CellProfiler 4 software to identify the nuclei (pre-stained with Hoechst 33342) and cytoplasm of each cell based on the fluorescence of the mClover protein (Fig. 1B). ERK activity in individual cells was calculated based on median mClover intensities in the nucleus and cytoplasm, and the cytoplasmic shape was used to measure the length of the cytoskeleton, including processes. This algorithm enabled the assessment of the changes in the mean ERK activity and the length of the cytoskeleton at each exposure, as well as a comparison of the ERK activity and changes in the cytoskeleton length in individual cells.

**ERK activity is associated with cell differentiation**

**Fig. 2:**
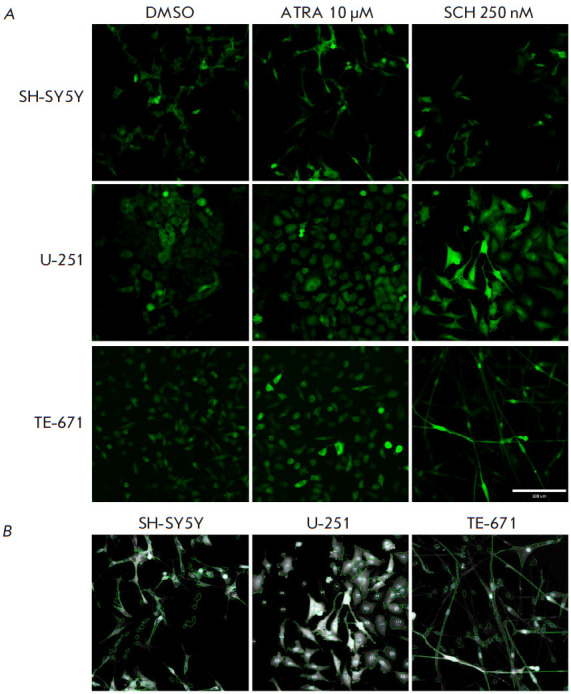
Changes in the ERK activity and cell length induced by retinoic acid and ERK inhibitors. (*A*) – Distributions of ERK activity (violin plots) in SH-SY5Y, U-251, and TE-671 cells 72 h after the addition of 10 μM retinoic acid (ATRA) or 250 nM of the ERK inhibitors SCH772982 (SCH), Ulixertinib (Ulix), and VX-11e. In controls, cells were added with DMSO because all agents were dissolved in DMSO. Each measurement included images of 6 independent, randomly selected fields (median values for each field are marked with dots). (*B*) – Cell length distributions (violin plots) in pixels 72 h after the addition of agents. Violin plots are based on the results of measuring ERK activity in at least 300 unique cells. Dots indicate median values for each of the 6 analyzed independent, randomly selected fields. Standard deviations (SDs) are shown in violin plots. The statistical significance is determined using the nonparametric Mann–Whitney U-test. (*C*) – Expression of the *PROM1 *(CD133), *RBFOX3 *(NeuN), *TUBB3 *(β3-tubulin), *MYOG *(myogenin), and *PAX7 *genes measured by real-time PCR 72 h after addition of agents. PCR data are normalized to the expression of the *GAPDH *gene in each sample; the results are presented as a logarithm of the change in gene expression relative to the control (DMSO-treated cells). Gene expression measurements were performed in triplicate. Plots show the mean expression change and 95% confidence interval. Statistical significance was determined using the Student’s t-test. (*D*) – ERK activity and cell length distributions in individual TE-671 cells 72 h after the addition of agents. *p-value < 0.05; **p-value < 0.01; ***p-value < 0.001

Exposure to retinoic acid led to a decrease in ERK activity in all three cell lines, SH-SY5Y, U-251, and TE-671. However, this decrease was less pronounced compared to the effect of the ERK inhibitors SCH772984, Ulixertinib, and VX-11e (Fig. 2A). In this case, retinoic acid induced process extension only in SH-SY5Y neuroblastoma cells. Induction of differentiation and, as a result, extension of cell processes are a well-known effect of retinoic acid on neuroblastoma cells. In turn, ERK inhibitors did not cause extension of the cytoskeleton in SH-SY5Y cells (Fig. 2B). Interestingly, we observed opposite effects in U-251 and TE-671 cells. For example, retinoic acid did not affect the length of TE-671 cells and even reduced the length of U-251 cells. In this case, ERK inhibitors, especially SCH772984, significantly increased the length of TE-671 and U-251 cells, in particular due to the extension of cell processes (Fig. 2B). It is also worth noting that SCH772984 was the most potent ERK inhibitor (Fig. 2A) and most strongly affected the length of U-251 and TE-671 cells.

To test whether the observed morphological changes were associated with cell differentiation, we measured the mRNA expression of the genes encoding differentiation markers (Fig. 2D). We measured the expression of the PROM1 gene, which encodes the CD133 protein, in all cells. Expression of this gene is characteristic of undifferentiated cells, in particular malignant neuroblastoma and glioblastoma stem cells and undifferentiated rhabdomyosarcoma cells. We also selected genes whose expression changes during neural cell differentiation: the RBFOX3 gene encoding the NeuN protein [26, 27] and the TUBB3 gene encoding β3-tubulin [27]. Because TE-671 rhabdomyosarcoma cells are known to be capable of differentiating into muscle cells, we chose the myogenin gene MYOG and the transcription factor gene PAX7 to analyze the differentiation of these cells [28]. Myogenin is one of the main markers of muscle cell differentiation, and PAX7 is an important regulator of early differentiation of these cells. We found a significant increase in the expression of the MYOG and PAX7 genes and a decrease in the expression of PROM1 in TE-671 cells exposed to SCH772984. Retinoic acid did not cause noticeable changes in the expression of these genes. There was also a slight decrease in PROM1 expression in U-251 cells treated with SCH772984 and a decrease in TUBB3 expression after exposure to retinoic acid. There were no significant changes in gene expression in SH-SY5Y cells. Interestingly, TE- 671 cells, whose length increased most substantially by SCH772984, had low ERK activity (Fig. 2D). These data indicate a relationship between ERK tyrosine kinase activity and differentiation of TE-671 cells at the level of both the entire population and individual cells.

**ERK inhibition alters the expression of growth factor receptors**

**Fig. 3:**
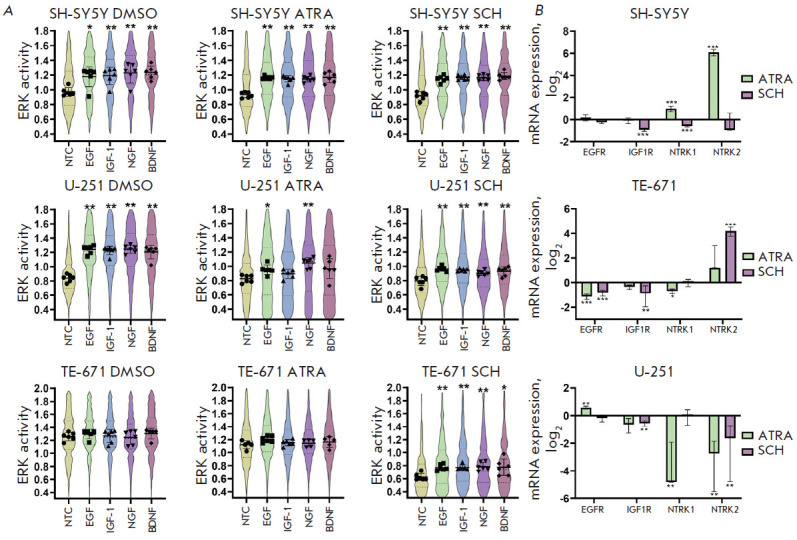
Effect of ERK inhibition and retinoic acid on growth factor activity. (*A*) – Distributions of ERK activity (violin plots) 30 min after addition of the growth factors EGF, IGF-1, NGF, and BDNF (100 ng/mL each) to SH-SY5Y, U-251, and TE-671 cells pretreated with 10 μM retinoic acid (ATRA) or 250 nM of the ERK inhibitor SCH772982 (SCH) for 72 h. DMSO-treated cells were used as the control. A non-treated control (NTC) is a cell added with the culture medium without growth factors. Dots indicate median values for each of 6 analyzed independent, randomly selected fields. Standard deviations (SDs) are shown in violin plots. Statistical significance is determined using the nonparametric Mann–Whitney U-test. (*B*) – Expression of the *EGFR*, *IGF-1R*, *NTRK1 *(*TrkA*), and *NTRK2 *(*TrkB*) genes by real-time PCR 72 h after the addition of agents. PCR data are normalized to the expression of the *GAPDH *gene in each sample; the results are presented as a logarithm of the change in gene expression relative to the control (DMSO-treated cells). Gene expression measurements were performed in triplicate. Plots show the mean expression change and a 95% confidence interval. Statistical significance is determined using the Student’s t-test. *p-value < 0.05; **p-value < 0.01; ***p-value < 0.001

How do the ERK inhibitor SCH772984 and retinoic acid affect cell sensitivity to growth factors? To understand this, we treated cells with the agents for 72 h, washed them from a culture medium containing the agents, and added a serum-free medium because the growth factors present in the serum could strongly affect ERK activity. Twelve hours after changing the medium, growth factors at a concentration of 100 ng/mL were added to the cells. We selected growth factors that are able to activate ERK in various cells, involved in neural or myogenic differentiation, and able to stimulate cell survival: epidermal growth factor (EGF) [29], insulin-like growth factor 1 (IGF-1) [30], neural growth factor (NGF) [15, 31], and brain-derived neurotrophic factor (BDNF) [15]. All growth factors significantly activated ERK in SH-SY5Y neuroblastoma cells, in particular after induction of cell differentiation with retinoic acid and treatment of cells with the ERK inhibitor SCH772984 (Fig. 3A). The growth factors also activated ERK in the control U-251 astrocytoma cells (treated with DMSO only) and SCH772984-treated cells (Fig. 3A). However, treatment of U-251 cells with retinoic acid resulted in less pronounced effects of EGF, IGF-1, and BDNF. There was no statistically significant effect of the growth factors on the control TE-671 cells and retinoic acid-treated cells. SCH772984-induced differentiation of TE-671 cells rendered the cells sensitive to all growth factors. It is important to note that undifferentiated TE-671 cells in the serum-free medium had high ERK activity comparable with that in the presence of serum, whereas basal ERK activity after treatment with the ERK inhibitor for 72 h was significantly lower. Probably, the initially high basal ERK activity of TE-671 cells prevents the detection of significant changes in the ERK activity induced by growth factors. However, even after prolonged exposure to the ERK inhibitor SCH772984, all cells either retained or acquired the ability to respond to growth factors (Fig. 3A).

Because the effect of growth factors can depend on both basal ERK activity and changes in the abundance of growth factor receptors during differentiation, we measured changes in the receptor mRNA expression. For this purpose, we chose the NTRK1 and NTRK2 genes encoding the main receptors of the used growth factors NGF and BDNF (TrkA and TrkB), IGF1R encoding the IGF-1 receptor, and EGFR encoding the EGF receptor. Expression of the NTRK2 gene, one of the main markers of neuroblastoma cell differentiation, was significantly upregulated in SH-SY5Y cells exposed to retinoic acid (Fig. 3B). We found similar changes in NTRK2 expression in TE-671 cells treated with SCH772984. Retinoic acid caused a slight increase in NTRK1 expression in TE-671 and SH-SY5Y cells. In U-251 cells, NTRK1 expression was significantly downregulated after treatment with retinoic acid, whereas NTRK2 expression was downregulated after treatment with both retinoic acid and SCH772984 (Fig. 3B). There were no significant changes in the EGFR and IGF1R expression. These data indicate that increased sensitivity of TE-671 cells to growth factors may be associated with both a decrease in the basal level of ERK activity and an increase in the expression of receptors; e.g., in the case of TrkA and TrkB.

## DISCUSSION

In this study, we investigated the effect of retinoic acid on the ERK activity and the effect of ERK inhibition on the differentiation of three types of malignant cells. We found a direct relationship between a decrease in the ERK activity in U-251 astrocytoma and TE-671 rhabdomyosarcoma cells and differentiation-associated morphological changes in these cells. Exposure of TE-671 cells to the ERK inhibitor SCH772984 for 72 h resulted in a significant increase in the expression of the myogenin gene MYOG. Increased myogenin expression is considered the main marker of skeletal muscle differentiation [32]. There was a decrease in the expression of the PROM1 gene that is typical of malignant stem cells. Changes in the expression of the PROM1 and MYOG genes and significant morphological changes in SCH772984-treated TE-671 cells indicate induction of myogenic differentiation. Our results are consistent with reported data holding that MEK inhibitors initiate the differentiation of rhabdomyosarcoma cells [22]. In this case, inhibition of MEK for 72 h led to a decrease in the expression of the PAX7 gene [28], whereas direct inhibition of ERK caused a significant increase in PAX7 expression. PAX7 is believed to be necessary for the initiation of myogenic differentiation, and its expression level is upregulated in early skeletal muscle progenitor cells [33]. At the later stages of differentiation, PAX7 expression usually decreases; however, there are no unambiguous data on the effect of changes in the PAX7 expression on cell differentiation upon suppression of ERK. The observed differences in PAX7 expression are possibly related to differences in MEK and ERK inhibition. There were no significant changes in the expression of the neural differentiation markers NeuN and β3-tubulin at the mRNA level in SH-SY5Y and U-251 cells treated with retinoic acid or the ERK inhibitor SCH772984. However, in SH-SY5Y cells, retinoic acid caused a significant increase in the expression of TrkB, the main differentiation marker in these cells. Interestingly, ERK inhibition-induced differentiation of TE-671 cells resulted in increased TrkB expression. This indicates a potential similarity in the regulation of TrkB expression in these cell types during differentiation. In the case of U-251 astrocytoma cells, differentiation markers suitable for a PCR analysis should be selected.

Using the ERK-KTR reporter system, we have shown that the length of TE-671 cells depends on a decrease in ERK activity both in the entire population and in individual cells. This indicates a direct relationship between ERK activity and cell differentiation. Similar results were obtained for U-251 astrocytoma cells. Retinoic acid-induced differentiation of SH-SY5Y neuroblastoma cells also led to a decrease in ERK activity. However, direct ERK inhibition in SH-SY5Y cells does not cause initiation of differentiation, which indicates a secondary role of the retinoic acid-induced decrease in ERK activity. Several studies have shown that ERK activation in neural stem cells or early progenitors initiates differentiation into neurons and suppresses differentiation into glial cells [34, 35]. It should be noted that astrocytomas [36] and rhabdomyosarcomas [37, 38] are characterized by mutations directly in the MAPK signaling cascade, which lead to ERK hyperactivation, whereas these mutations are relatively rare in neuroblastomas. TE-671 cells contain a mutation in the NRAS gene (Q61H) [39, 40], which leads to ERK hyperactivation, and U-251 cells contain a deletion in the NF1 gene [41] that encodes neurofibromin, a negative regulator of RAS proteins and the RAS-MEK-ERK signaling cascade [42]. The F1174L mutation in ALK receptor tyrosine kinase also leads to ERK activation in SH-SY5Y cells, but it does not directly regulate the RAS-MEK-ERK signaling cascade. Probably, mutations in the RAS-MEK-ERK cascade are responsible for the initiation of the differentiation induced by ERK inhibition in TE-671 and U-251 cells [40].

Our study has several limitations. Although we see morphological changes and changes in gene expression, which are induced by ERK inhibitors, we cannot identify the stage to which cell differentiation proceeds. In addition, it is not known whether TE-671 cells can differentiate into normal muscle cells upon ERK inhibition. However, ERK inhibition can be at least an initiating event that does not suppress the activity of growth factors. Also, it cannot be unambiguously asserted that the observed morphological changes in U-251 cells are differentiation. Therefore, additional experiments are required, in particular a search for reliable markers of differentiation.

## CONCLUSION

In this study, we have established a relationship between morphological changes associated with the differentiation of TE-671, U-251, and SH-SY5Y cells and the activity of ERK kinases, in particular at the level of individual cells. We have demonstrated that ERK inhibition in TE-671 rhabdomyosarcoma cells initiates their myogenic differentiation. Differentiation renders TE-671 cells more sensitive to growth factors, potentially by reducing basal ERK activity and increasing TrkB expression. Our findings can be used to develop new protocols for cell differentiation: in particular, for basic research and the development of new approaches to the therapy of malignant diseases.

## Abbreviations

ATRA: all-trans retinoic acid (tretinoin)
ERK: extracellular signal-regulated kinase
NGF: nerve growth factor
BDNF: brain-derived neurothropic factor
EGF: epidermal growth factor
IGF-1: insulin-like growth factor 1
EGFR: epidermal growth factor receptor
IGF1R: insulin-like growth factor 1 receptor
TrkA: tropomyosin receptor kinase A
TrkB: tropomyosin receptor kinase B
MYOG: myogenin
FBS: fetal bovine serum

## References

[1] Lavoie H., Gagnon J., Therrien M. (2020). Nat. Rev. Mol. Cell. Biol..

[2] Orlova N.N., Lebedev T.D., Spirin P.V., Prassolov V.S. (2016). Mol. Biol. (Moskow)..

[3] Bonni A., Brunet A., West A.E., Datta S.R., Takasu M.A., Greenberg M.E. (1999). Science..

[4] Spirin P., Lebedev T., Orlova N., Morozov A., Poymenova N., Dmitriev S.E., Buzdin A., Stocking C., Kovalchuk O., Prassolov V. (2017). Oncotarget..

[5] Ma M., Bordignon P., Dotto G.P., Pelet S. (2020). Heliyon..

[6] Simon C.S., Rahman S., Raina D., Schroter C., Hadjantonakis A.K. (2020). Dev. Cell..

[7] Pokrass M.J., Ryan K.A., Xin T., Pielstick B., Timp W., Greco V., Regot S. (2020). Dev. Cell..

[8] Ogura Y., Sami M.M., Wada H., Hayashi S. (2019). Genes Cells..

[9] Maryu G., Matsuda M., Aoki K. (2016). Cell Struct. Funct..

[10] Hamilton W.B., Mosesson Y., Monteiro R.S., Emdal K.B., Knudsen T.E., Francavilla C., Barkai N., Olsen J.V., Brickman J.M. (2019). Nature.

[11] Spelat R., Ferro F., Curcio F. (2012). J. Biol. Chem..

[12] Ying Q.L., Wray J., Nichols J., Batlle-Morera L., Doble B., Woodgett J., Cohen P., Smith A. (2008). Nature.

[13] Tamama K., Sen C.K., Wells A. (2008). Stem Cells Dev..

[14] Yu Y., Wang X., Zhang X., Zhai Y., Lu X., Ma H., Zhu K., Zhao T., Jiao J., Zhao Z.A. (2018). Stem Cell Res. Ther..

[15] Liu F., Xuan A., Chen Y., Zhang J., Xu L., Yan Q., Long D. (2014). Mol. Med. Rep..

[16] Yan L., Zhou L., Yan B., Zhang L., Du W., Liu F., Yuan Q., Tong P., Shan L., Efferth T. (2020). Cell Death Dis..

[17] Khavari T.A., Rinn J. (2007). Cell Cycle..

[18] Kern F., Niault T., Baccarini M. (2011). Br. J. Cancer..

[19] Olson E.N., Spizz G., Tainsky M.A. (1987). Mol. Cell. Biol..

[20] Reynolds C.P., Matthay K.K., Villablanca J.G., Maurer B.J. (2003). Cancer Lett..

[21] Tallman M.S., Andersen J.W., Schiffer C.A., Appelbaum F.R., Feusner J.H., Ogden A., Shepherd L., Willman C., Bloomfield C.D., Rowe J.M. (1997). N. Engl. J. Med..

[22] Yohe M.E., Gryder B.E., Shern J.F., Song Y.K., Chou H.C., Sindiri S., Mendoza A., Patidar R., Zhang X., Guha R. (2018). Sci. Transl. Med..

[23] Forshew T., Tatevossian R.G., Lawson A.R., Ma J., Neale G., Ogunkolade B.W., Jones T.A., Aarum J., Dalton J., Bailey S. (2009). J. Pathol..

[24] Tanaka T., Higashi M., Kimura K., Wakao J., Fumino S., Iehara T., Hosoi H., Sakai T., Tajiri T. (2016). J. Pediatr. Surg..

[25] Regot S., Hughey J.J., Bajar B.T., Carrasco S., Covert M.W. (2014). Cell..

[26] Gusel’nikova V.V., Korzhevskiy D.E. (2015). Acta Naturae..

[27] Constantinescu R., Constantinescu A.T., Reichmann H., Janetzky B. (2007). J. Neural. Transm. Suppl..

[28] De Luna N., Suarez-Calvet X., Garicano M., Fernandez-Simon E., Rojas-Garcia R., Diaz-Manera J., Querol L., Illa I., Gallardo E. (2018). J. Neuropathol. Exp. Neurol..

[29] Garcez R.C., Teixeira B.L., Schmitt Sdos S., Alvarez-Silva M., Trentin A.G. (2009). Cell Mol. Neurobiol..

[30] Galvin C.D., Hardiman O., Nolan C.M. (2003). Mol. Cell. Endocrinol..

[31] Lebedev T.D., Vagapova E.R., Popenko V.I., Leonova O.G., Spirin P.V., Prassolov V.S. (2019). Front. Oncol..

[32] Bentzinger C.F., Wang Y.X., Rudnicki M.A. (2012). Cold Spring Harb. Perspect. Biol..

[33] Florkowska A., Meszka I., Zawada M., Legutko D., Proszynski T.J., Janczyk-Ilach K., Streminska W., Ciemerych M.A., Grabowska I. (2020). Stem Cell Res. Ther..

[34] Paquin A., Barnabe-Heider F., Kageyama R., Miller F.D. (2005). J. Neurosci..

[35] Samuels I.S., Karlo J.C., Faruzzi A.N., Pickering K., Herrup K., Sweatt J.D., Saitta S.C., Landreth G.E. (2008). J. Neurosci..

[36] Jones D.T., Gronych J., Lichter P., Witt O., Pfister S.M. (2012). Cell Mol. Life Sci..

[37] Marampon F., Bossi G., Ciccarelli C., Di Rocco A., Sacchi A., Pestell R.G., Zani B.M. (2009). Mol. Cancer Ther..

[38] Ciccarelli C., Vulcano F., Milazzo L., Gravina G.L., Marampon F., Macioce G., Giampaolo A., Tombolini V., Di Paolo V., Hassan H.J. (2016). Mol. Cancer..

[39] Stratton M.R., Darling J., Pilkington G.J., Lantos P.L., Reeves B.R., Cooper C.S. (1989). Carcinogenesis..

[40] Dolgikh N., Hugle M., Vogler M., Fulda S. (2018). Cancer Research.

[41] McGillicuddy L.T., Fromm J.A., Hollstein P.E., Kubek S., Beroukhim R., De Raedt T., Johnson B.W., Williams S.M., Nghiemphu P., Liau L.M. (2009). Cancer Cell..

[42] Cichowski K., Jacks T. (2001). Cell..

